# A Membrane-Bound Vertebrate Globin

**DOI:** 10.1371/journal.pone.0025292

**Published:** 2011-09-20

**Authors:** Miriam Blank, Jessica Wollberg, Frank Gerlach, Katja Reimann, Anja Roesner, Thomas Hankeln, Angela Fago, Roy E. Weber, Thorsten Burmester

**Affiliations:** 1 Biocenter Grindel, University of Hamburg, Hamburg, Germany; 2 Institute of Zoology, Johannes-Gutenberg-University of Mainz, Mainz, Germany; 3 Institute of Molecular Genetics, Johannes-Gutenberg-University, Mainz, Germany; 4 Zoophysiology, Department of Biological Sciences, Aarhus University, Aarhus, Denmark; University of South Florida College of Medicine, United States of America

## Abstract

The family of vertebrate globins includes hemoglobin, myoglobin, and other O_2_-binding proteins of yet unclear functions. Among these, globin X is restricted to fish and amphibians. Zebrafish (*Danio rerio*) globin X is expressed at low levels in neurons of the central nervous system and appears to be associated with the sensory system. The protein harbors a unique N-terminal extension with putative N-myristoylation and S-palmitoylation sites, suggesting membrane-association. Intracellular localization and transport of globin X was studied in 3T3 cells employing green fluorescence protein fusion constructs. Both myristoylation and palmitoylation sites are required for correct targeting and membrane localization of globin X. To the best of our knowledge, this is the first time that a vertebrate globin has been identified as component of the cell membrane. Globin X has a hexacoordinate binding scheme and displays cooperative O_2_ binding with a variable affinity (*P*_50_∼1.3–12.5 torr), depending on buffer conditions. A respiratory function of globin X is unlikely, but analogous to some prokaryotic membrane-globins it may either protect the lipids in cell membrane from oxidation or may act as a redox-sensing or signaling protein.

## Introduction

Globins are small heme-proteins that have the ability to reversibly bind molecular oxygen (O_2_). For a long time only two globin types have been known in vertebrates: hemoglobin (Hb) and myoglobin (Mb). Most likely, Hb and Mb are the best-studied proteins in biological, biochemical, biophysical and medical sciences. Hb, which resides in the cytoplasm of red blood cells and serves to transport O_2_ from the respiratory organs to the tissue, is certainly the best known globin type [1]. Vertebrate Hb is a tetramer composed of two α- and two β-chains. Mb is a monomer that is mainly located in the cytoplasm of the myocytes in heart or skeletal muscles. Mb stores O_2_, facilitates O_2_ diffusion to the mitochondria, and may also be involved in the decomposition of NO [2].

Within the past ten years, sequencing of expressed sequence tags (ESTs) and whole genomes revealed the presence of additional globin types in vertebrates, such as neuroglobin (Ngb) and cytoglobin (Cygb). Ngb resides in the central and peripheral nervous system of vertebrates [3]. The exact function of Ngb is still uncertain [4], [5], but there is evidence that it has a protective role related to the oxidative metabolism [6], [7]. Cygb is expressed in fibroblast-related cell types and distinct neurons [8], [9], [10], [11]. Cygb is possibly involved in collagen synthesis or O_2_ supply to distinct enzymes [5], [11]. Deoxygenated Hb and Mb display a so-called pentacoordinated heme, while Ngb and Cygb are hexacoordinated [9], [12].

Ngb and Cygb are widespread among vertebrates and occur in fishes, amphibians, reptiles, birds and mammals [13]. Other globins appear to be restricted to certain vertebrate classes. In birds, an eye-specific globin has been identified [14], [15], while globin Y is expressed in various tissues of *Xenopus* [16].

GbX has only been identified in “lower” vertebrates, *i.e.* fishes and amphibians, but appears to have been lost in Amniota [17]. Although the GbX sequence is highly conserved among fishes and amphibians, it displays only limited similarities to any other globin. In phylogenetic analyses GbX joins a clade consisting of Ngb, invertebrate nerve globins and the Hbs of the tunicate *Ciona intestinalis.* GbX is widely expressed in goldfish tissues [17] but displays a more restricted localization in brain and eye of *Xenopus* [16]. Hypoxia decreases the levels of GbX mRNA in adult zebrafish [18]. Due to unique N- and C-terminal extensions, the GbX sequence is longer than that of a typical globin (∼150 amino acids) and spans ∼200 amino acids.

Our results indicate that GbX is anchored in the cell-membrane by dual N-terminal acylation. While membrane-bound globins have previously been identified in some bacteria [19] and the green shore crab (*Carcinus maenas*) [20], GbX is the first such globin-type discovered in vertebrates. In order to gain further insight into possible functional role of GbX, we also investigated heme coordination and reactivity towards O_2_ and the ability to form disulfide bonds. Our data provide evidence for a novel globin function in vertebrates that is associated with its membrane localization and unlikely to be involved O_2_ transport and storage.

## Methods

### RNA extraction and cDNA cloning

Experiments with zebrafish were approved by the Hamburg authorities (*Behörde für Gesundheit und Verbraucherschutz*, License No. 57/07). Total RNA was obtained from adult zebrafish tissues using the RNeasy Mini-Kit (Qiagen, Hilden, Germany). 30 mg tissue samples were supplemented with 600 µl RLT buffer and further purified using the silica column method according to the manufacturer's instructions (Qiagen). The quality and integrity of RNA was evaluated photometrically and by agarose gel electrophoresis. The coding sequence of zebrafish GbX was isolated by reverse-transcription PCR from total RNA. To ensure maximal expression in the recombinant system, the N- and C-termini were removed by application of appropriate primers. The resulting GbXΔNC cDNA fragment, covering amino acids 25 to 184, of *D. rerio* GbX was cloned into the pET3a vector.

### Recombinant expression and purification of globin X

GbXΔNC was expressed in *E. coli* BL21(DE3)pLysS host cells (Stratagene, Heidelberg, Germany). *E. coli* were grown over night at 30°C in L-medium (1% bactotryptone, 0.5% yeast extract, 0.5% NaCl, pH 7.5) with 10 µg/ml ampicillin, 34 µg/ml chloramphenicol. 3 ml of this culture was applied to 500 ml L-medium supplemented with 1 mM δ-amino-levulinic acid. The culture was induced at OD_600_ = 0.4 to 0.8 by the addition of isopropyl-thio-D-galactopyranoside to a final concentration of 0.4 mM. Expression was continued at 27°C over night. Cells were harvested (45 min centrifugation at 4.000 x g) and resuspended in 20 ml lysis buffer (50 mM Tris-HCl, pH 8.0; 1 mM MgCl_2_; 1 mM dithiotreitol), supplemented with 10 µg/ml DNase, 5 µg/ml RNase, Complete™ proteinase inhibitor mix (Roche Applied Science, Mannheim, Germany) and 1 mM Pefabloc (Roth, Karlsruhe, Germany). The cells were broken by three freeze-thaw cycles in liquid nitrogen followed by ultrasonication (10×30 s). The sample was incubated for 2 h at 37°C to digest the DNA and RNA. The cell debris was removed by centrifugation for 1 h at 4°C at 5,500×g. The supernatant was fractionated by ammonium sulfate precipitation. The reddish 70–80% ammonium sulfate pellet was dissolved in 5 mM Tris-HCl, pH 8.5, and desalted using an Amicon Ultra filter (Millipore, Schwalbach, Germany). Further purification of GbX was achieved by a HiPrep™ 16/10 Q XL ion-exchange column (GE Healthcare, Freiburg, Germany) with a gradient of 0 to 1 M NaCl in 20 mM Tris-HCl, pH 8.5. Size exclusion chromatography was carried out using a HiLoad™ 16/60 Superdex™ 75 prep grade column (GE Healthcare). The final GbX fractions were analyzed by gel electrophoresis, pooled, concentrated, and stored at −20°C. Protein concentrations were determined according to Bradford [21].

### Gel electrophoresis

Protein extracts in sample buffer (31.25 mM Tris-HCl, pH 6.8, 1% SDS, 2.5% β-mercaptoethanol, 5% glycerol) were heat-denatured for 5 minutes at 95°C and loaded to a 15% SDS-PAGE. GbX was further analyzed using a PhastSystem (GE Healthcare) on ultrathin polyacrylamide gels by isoelectric focusing (IEF) in the 3–9 pH range under native conditions and by SDS-PAGE in 10–15% gradient gels in the absence and presence of DTT in the sample buffer.

### Matrix-Assisted Laser Desorption/Ionization Time-Of-Flight Mass Spectrometry (MALDI-TOF)

MALDI-TOF experiments were performed on recombinant proteins that had been purified by chromatography as described above as well as on recombinant proteins that had been separated by SDS-PAGE before in-gel digestion with trypsin. The MALDI-TOF data were evaluated using the program MASCOT (Matrix Science, London, UK).

### O_2_ equilibria

The recombinant truncated form of purified *D. rerio* GbX (GbXΔNC) was converted into the ferrous derivative by incubating the protein in a closed anaerobic vial containing the enzymatic reducing system [22], as described previously for Ngb and Cygb [23], on ice for >1 h, in the presence or absence of 2.5 mM DTT.

O_2_ binding equilibria of recombinant truncated GbX (4 µl samples) were measured with the enzymatic reducing system present. Experiments were carried out at 20°C using a thin-layer equilibration chamber connected to cascaded Wösthoff gas-mixing pumps that deliver continuous flow of precise mixtures of N_2_ and O_2_, and provides a stepwise increase in O_2_ tension (*P*O_2_). Equilibration at each step was monitored as absorbance changes at 428 nm (maximum absorption of deoxyHb) or in the visible spectrum (400–700 nm) using a UV-visible Cary 50 Probe spectrophotometer equipped with optic fibers [23]. Reversibility was checked by measuring a spectrum (400–620 nm) after equilibration with pure N_2_ and O_2_ at the end of each experiment. Samples were adjusted to a final protein concentration of 0.1 mM heme in 0.1 M HEPES buffer, 0.5 mM EDTA, in the presence of the enzymatic reducing system [22] and in the presence and absence of 2.5 mM DTT. O_2_ affinity (P_50_, oxygen tension at half-saturation) and cooperativity (n_50_) were interpolated from the zero-intercept and the slope, respectively, of Hill plots, log[Y/(1-Y)] versus log *P*O_2_, where Y is the O_2_ fractional saturation.

### Quantitative real-time RT-PCR (qRT-PCR)

Total RNA was extracted from tissues (eye, brain, heart, gills, muscle, liver) using PeqGold TriFast (Peqlab, Erlangen, Germany) in combination with the RNeasy Mini Kit (Qiagen), according to the manufacturer's instructions with an additional DNase I digestion step. The integrity of RNA was verified by gel electrophoresis and reading the 260/280 nm absorption ratio. Reverse transcription was carried out with 770 ng total RNA in a 20 µl reaction using the SuperScript III reverse transcriptase (Invitrogen, Darmstadt, Germany). QRT-PCR amplification was performed on the ABI Prism 7300 Real Time PCR System (Applied Biosystems, Darmstadt, Germany). PCR reactions were performed in triplicates in 25 µl, including the Power SYBR Green PCR Master Mix, 3 µl of cDNA and primers (final concentration 0.2 µM). Primer sequences were 5′-GACTCATCCAGAGTGTAAAGATGT-3′ and 5′-GAGTCTCTAAACGCTCCAGC-3′. The *Taq* DNA polymerase was activated at 95°C for 10 min and amplification was carried out using a standard PCR protocol (95°C for 15 s, 60°C for 15 s, and 72°C for 30 s; 40 cycles) with fluorescence measurement at the last step of each cycle. Dissociation curve analysis was used to validate the specificity of each amplification reaction. Absolute mRNA copies were calculated with the 7300 System Sequence Detection Software 1.3.1 (Applied Biosystems) by means of the standard curve method by using dilutions (10^6^ to 10^2^) of the recombinant plasmid.

### Antibody preparation

A polyclonal antibody against *D. rerio* GbX was produced by a commercial service (Eurogentec, Seraing, Belgium). The antibody was raised in rabbits against recombinant GbXΔNC. Specific anti-GbX antibody was affinity-purified using GbXΔNC coupled to a SulfoLink column (Pierce, Fisher Scientific, Bonn Germany) according to the manufacturer's instructions and stored in 50 mM Tris, 100 mM glycine, pH 7.4) supplemented with 0.1% NaN_3_.

### Immunohistochemistry

Tissues were fixed for 2 h in 4% paraformaldehyde in PBS (140 mM NaCl, 2.7 mM KCl, 8.1 mM Na_2_HPO_4_, 1.5 mM KH_2_PO_4_) and stored in PBS at 4°C until use. Cryosections of 12 to 16 µm thickness were placed on silanized coverslips and air-dried for 2 h. Non-specific binding sites were blocked for 15 min in PBS/0.1% Triton X-100/1% bovine serum albumin. Anti-GbXΔNC antibodies were diluted 1∶100 in PBS/0.1% Triton X-100/1% bovine serum albumin overnight at room temperature. The sections were washed three times 8 min in PBS, incubated for 2 h with donkey anti-rabbit F(ab)_2_-fragment coupled to Cy3 (1∶500 in PBS; Dianova, Hamburg, Germany) and embedded in 1x PBS/glycerol. The Hoechst dye 33258 (0.3 µg/ml) was added to stain the nuclei. Sections were analyzed by an Olympus BX51 microscope. Images were merged and labeled using Adobe Photoshop CS4.

### Transient expression of GbX in cell culture

The full length (GbX) and C-terminally truncated GbX (GbXΔC) sequences of *D. rerio* were amplified from zebrafish cDNA by application of appropriate primers providing the XhoI and EcoRI restriction site and a Kozak sequence. cDNAs were cloned in-frame in the pEGFP-N1 vector (a kind gift from B. Gellersen) to produce a GbX protein C-terminally tagged with enhanced green fluorescence protein. Three mutant constructs of GbXΔC were generated using the same approach. Non-myristoylatable mutant of GbXΔC was constructed by changing Gly at amino acid position 2 to Ala (GbX-G2A) and a non-palmitoylatable mutant (GbX-C3S) was generated by changing amino acid position 3 from Cys to Ser using appropriate primers. For construction of a non-acylatable mutant (GbX-AS) both the myristoylation and palmitoylation site are inactivated. Mutated constructs were confirmed by sequencing (GATC Biotech).

Mouse fibroblast (3T3) were obtained from the American Type Culture Collection (ATCC, Manassas, USA). Cells were grown on 8-well coverslips and transfected at ∼70% confluency with 0.5 µg plasmid DNA per well using Nanofectin (PAA, Cölbe, Germany) according to the manufacturer's instructions. Cells were grown at 37°C and 5% CO_2_ in Dulbecco's modified eagle's medium in the presence of G418 (Invivogen, Toulouse, France) for two days after transfection. Expression of fusion proteins was verified by Western blotting. Transiently transfected cells were harvested in RIPA buffer (50 mM Tris-HCl, pH 7.4, 150 mM NaCl, 1% Triton X-100 0.5% sodium deoxycholate, 0.1% SDS) and 10 µg (GFP plasmid) and 40 µg (constructs) protein were denatured in sample buffer for 5 min at 95°C. Proteins were separated by a SDS-polyacrylamide gel and transferred to a PVDF membrane (Biorad, Munich, Germany). Non-specific binding sites were blocked for 1 h with 1% nonfat dry milk/TBS. Primary anti-GFP antibody (Abcam, Berlin, Germany) was diluted 1∶1000 in 1% nonfat dry milk/TBS and immunodetection was performed over night at 4°C. After washing, the membrane was incubated with an anti-rabbit antibody coupled with horseradish peroxidase (1∶10,000 in TBS, 1.5 h at room temperature). Detection was carried out using enhanced chemiluminescence (Peqlab).

Slides were washed in PBS and cells were fixed using 4% (w/v) paraformaldehyde in PBS for 10 min and nonspecific sites were blocked with 1% (w/v) BSA in PBS for 2 hours. Primary antibody anti-GbXΔNC was diluted in 1% BSA / 0.4% Triton-X-100 / PBS to a final concentration of 1∶500 and cells were incubated overnight at 4°C. Slides were then incubated with a Cy3 conjugated anti-rabbit F(ab)_2_-fragment secondary antibody (1∶1000 in PBS; Dianova) for 2 hours. Afterwards Hoechst dye 33258 (1∶1000 in PBS) was applied to the cells for 15 min. Coverslips were washed three times in PBS for 5 min between each step. Finally, cells were mounted on the slides in Mowiol 4–88 (Roth, Karlsruhe, Germany) in combination with the anti-bleaching reagent 1.4-Diazabicyclo-(2.2.2)octan (Roth). Images were acquired on an Olympus BX51 microscope and combined using Adobe Photoshop CS.

### Bioinformatics

The tools provided by the ExPASy Molecular Biology Server of the Swiss Institute of Bioinformatics (http://www.expasy.ch) were used for sequence analyses. Myristoylation- and palmitoylation-sites were predicted by the myristoylator [24] and CSS-Palm 2.0 [25] respectively. PSORT II was used to predict subcellular localization [26].

## Results

### Recombinant *D. rerio* GbX

Recombinant expression of full-length *D. rerio* GbX in *E. coli* resulted in the formation of insoluble inclusion bodies. To overcome this problem, an N- and C-terminally truncated version of GbX, spanning amino acids 25 to 184 and thus the globin core, was generated (GbXΔNC). SDS-PAGE of purified GbXΔNC showed a protein of ∼18 kDa (Figure S1). A second, minor band of ∼36 kDa suggests the formation of a stable dimer (see below). The identity of the proteins was verified by MALDI-TOF, which identified GbX in both the monomer and the dimer after tryptic digest.

### Heme ligation and oxygen binding of GbX

**Figure 1:**
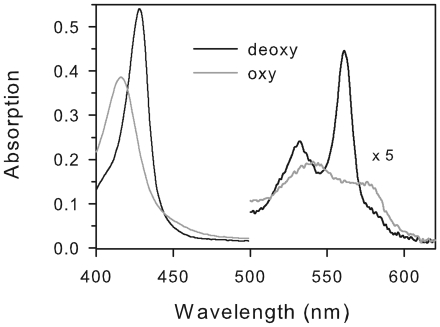
Absorbance spectra of zebrafish GbX. Spectra were obtained from N_2_- (deoxy) and O_2_-equilibrated 4-µl samples of recombinant GbX in the presence of the enzymatic reducing system at pH 7.8. Spectra measured using a UV-visible Cary 50 Probe spectrophotometer equipped with optic fibers.

Heme coordination of recombinant *D. rerio* GbXΔNC was investigated by measuring the absorption spectra from 360 to 600 nm in the absence or presence of sodium dithionite. The spectrum measured in air before heme reduction is typical of hexacoordinate ferric (Fe^3+^) heme (with a broad peak centered at 532 nm). The deoxy-spectrum (peaks at 428, 532 and 561 nm) was similar to those reported for Ngb and Cygb [12], [23], indicating that this globin is hexacoordinate in the absence of external ligands (Figure 1). Before measurements of O_2_ equilibrium curves, the oxy and deoxy spectrum were measured after equilibration with pure O_2_ and N_2_ respectively of 4-µl samples previously reduced to the ferrous form. The oxy spectrum (peaks at 416, 541 and 576 nm) did not change significantly over time after removal of the reducing system by gel filtration (not shown).

**Figure 2:**
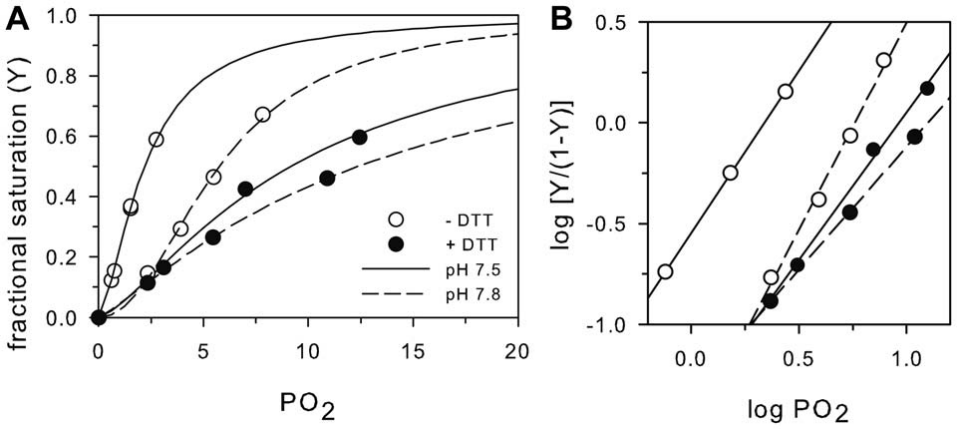
O_2_-binding kinetics of GbX. O_2_ equilibrium curves (A) and Hill plots (B) in the presence (closed circles) and absence (open triangles) of 2.5 mM DTT, at pH 7.5 (sold line) and 7.8 (discontinuous line). Temperature, 20°C, buffer, 0.1 M HEPES, protein concentration 0.1 mM heme. Other details are explained in the “Experimental” section.

O_2_ equilibrium curves showed that GbXΔNC has a variable O_2_ affinity. At 20°C and pH 7.4, P_50_ was between ∼1.3–12.5 torr, depending on pH and the presence of DTT (Figure 2A). DTT was added to investigate the potential role of reactive Cys on heme reactivity, as previously done for Ngb and Cygb [23], [27]. DTT increases P_50_ approximately fourfold at pH 7.5 and twofold at pH 7.8, revealing a role of disulfide bridge formation in the control of heme reactivity. Variable cooperativity coefficients (n_50_) of ∼1.2–2.1 (Figure 2B) indicate formation of a multisubunit complex, possibly a dimer or a teramer, independently of the presence of DTT. A decrease in pH results in an increase in the O_2_ affinity (i.e. decrease in P_50_, Figure 2A), both in the absence and in the presence of DTT (ΔlogP_50_/ΔpH∼0.6). As proposed for Ngb [23], this effect may originate from an open heme pocket (that provides greater access to external ligands) resulting from protonation of the distal His that may swing out of the pocket.

SDS-PAGE in the absence and presence of DTT showed that disulfide bond formation is intra- rather than inter-molecular (Figure S2). In the absence of DTT, a band reflecting a lower molecular mass appears, consistent with the formation of an internal disulfide bond and a more compact structure of the polypeptide chain. Formation of a dimeric structure, as evident from a weak band at ∼36 kDa, occurs to a lower extent. Isoelectrofocusing on polyacrylamide gels of the same sample indicated isoelectric points of the native protein of 7.5 and 7.8, which appear to be values for the protein with and without the internal disulfide bond, respectively, and is supported by the higher isoelectric point occurring after prolonged incubation under air (Figure S2).

### GbX in zebrafish brain and eye

**Figure 3:**
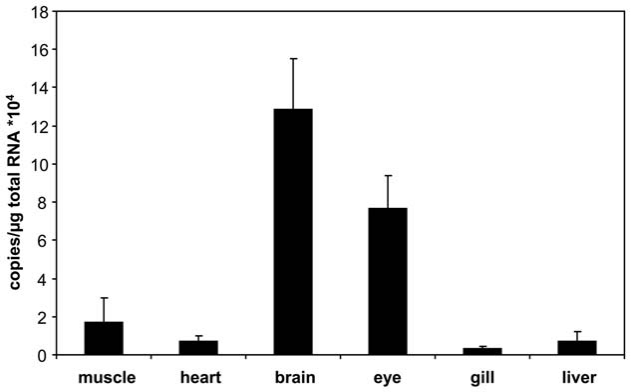
Expression of GbX in zebrafish. GbX mRNA levels were measured by quantitative real-time RT-PCR. Tissues of three individuals were pooled. Values are means of three independent pools and the standard deviations are given.

**Figure 4:**
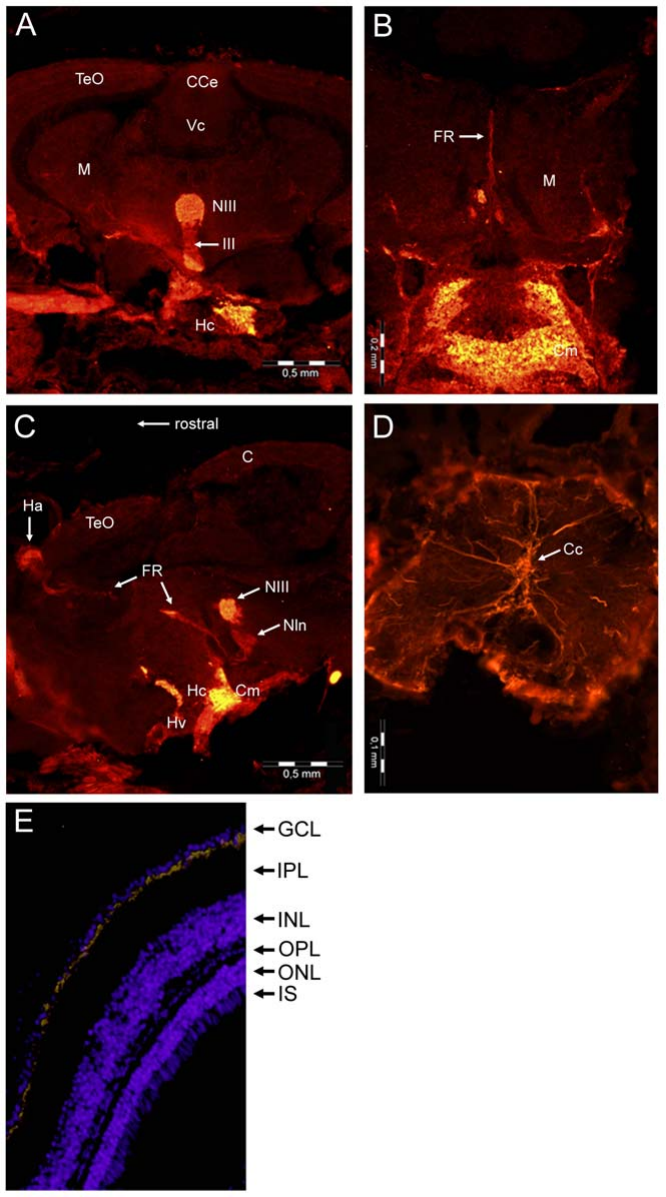
Localization of GbX in the central nervous system. Frontal section of the zebrafish brain stained with anti-GbX antibodies (A), showing the mesencephalon (M) with the tectum opticum (TeO) and the cerebellar corpus (CCe) and valvula cerebelli (Vc), two parts of the cerebellum. Staining is visible in the oculomotor nucleus (NIII) and the *nervus oculomotorius* (III) as well as in the caudal zone of periventricular hypothalamus (Hc). Higher magnification of stained regions of the hypothalamus (ventral) with *corpus mamillare* (Cm) and mesencephalon (dorsal) with *fasciculus retroflexus* (FR) (B). Sagittal section of the immunoreactive parts of the brain (C) with habenula (Ha), interpeduncular nucleus (Nln) and ventral zone of periventricular hypothalamus (Hv). Cross section of the spinal cord (D) showing staining in the spinal nerves of the dorsal and ventral roots. Localization of GbX in the ganglion cell layer of zebrafish retina (E). Cross section of the eye stained with the anti-GbX antibody (yellow). For staining of nuclei Hoechst 33258 was used. Ganglion cell layer (GCL), inner (IPL) and outer plexiform layer (OPL), inner (INL) and outer nuclear layer (ONL) and inner photoreceptor segment (IS).

qRT-PCR revealed the highest levels of GbX mRNA in the brain and eye of *D. rerio* (Figure 3). This was confirmed by immunofluorescence studies, which showed no detectable anti-GbX immunoreactivity in tissues other than the brain and eye of the zebrafish. The specificity of the GbX antibody was verified by pre-absorption tests using purified recombinant GbX protein. Strong GbX immunoreactivity was found in the mesencephalon, especially in the oculomotor nucleus (NIII) and oculomotor nerve (Figure 4A). In contrast, no staining was observed in the other parts of the mesencephalon, the cerebellum (cerebellar corpus and *valvula cerebelli*) and in the *tectum opticum*, which is the most prominent part of fish brain. Additionally, immunostaining was observed in the hypothalamic *corpus mamillare*, which is located ventrally (Figure 4B). Sagittal sections revealed a more detailed view of diencephalic staining. Strong signals were found in the caudal zone of the periventricular hypothalamus and the *fasciculus retroflexus* that projects to the interpeduncular nucleus (Figure 4C). In sections of the spinal cord, GbX was found in the spinal nerves of the dorsal and ventral roots (Figure 4D). The localization of GbX was further analyzed in zebrafish retina. In cross-sections of the eye, specific GbX labeling was detected in the ganglion cell layer (Figure 4E).

### Subcellular localization of GbX

**Figure 5:**
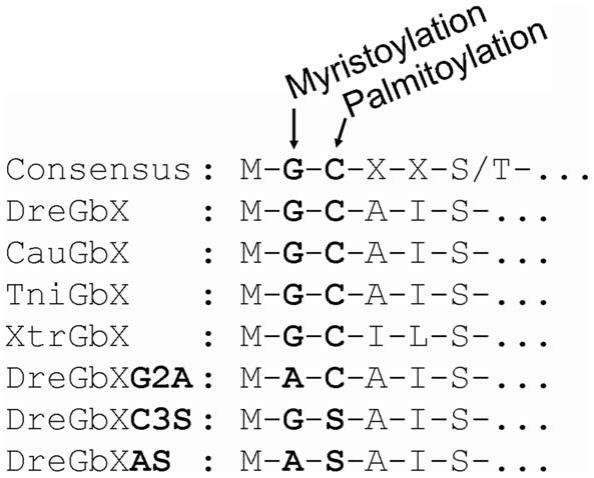
Myristoylation and palmitoylation of globin X. Alignment of the consensus motif for myristoylation and palmitoylation (upper row) and amino acid sequences of the GbX N-terminal extensions of low vertebrate species. Predicted sites are in bold print. DreGbX: *Danio rerio* globin X, CauGbX: *Carassius auratus* globin X: TniGbX: *Tetraodon nigroviridis* globin X, XtrGbX: *Xenopus tropicalis* globin X. Mutants of *D. rerio* globin X: non-myristoylatable mutant (DreGbXG2A), non-palmitoylatable mutant (DreGbXC3S), non-acylatable mutant (DreGbXAS).

**Figure 6:**
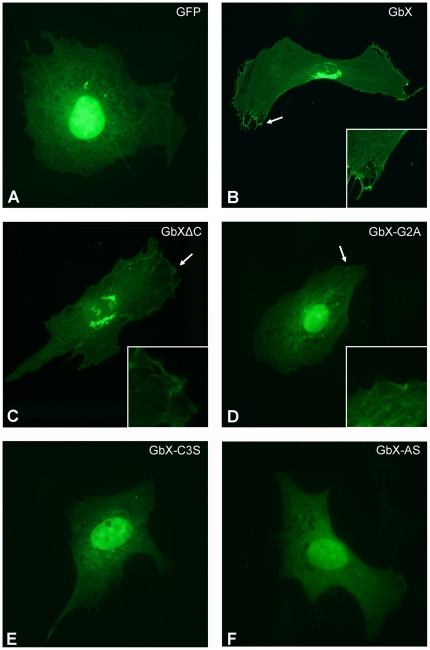
Membrane-localization of GbX. 3T3 cells were transiently transfected with GbX fusion constructs C-terminally tagged with GFP. GFP: EGFP vector without GbX, GbX: full length GbX, GbXΔC: C-terminally truncated GbX, GbX-G2A: non-myristoylatable mutant, GbX-C3S: non-palmitoylatable mutant, GbX-AS: non-acylatable mutant. Insets: Higher magnification of membrane-bound GbX.

Bioinformatic analyses identified signatures for dual fatty acid acylation at the N-termini of all known GbX sequences. An N-myristoylated Gly was predicted at position 2 and a putative S-palmitoylation site was found at Cys at position 3 (Figure 5). No other transport or signaling motifs were identified. To further investigate the intracellular localization and the function of N-terminal acylation of GbX, we generated GbX-GFP fusion constructs, which were modified by site-directed mutagenesis. Expression of fusion constructs was verified by Western blotting using an anti-GFP antibody. In all experiments, the fusion protein was detected with the expected masses of 50 (GbX) and 47 kDa (GbXΔC, G2A, C3S, AS), respectively (Figure S3). Fluorescence microscopy revealed that transfection with GFP-containing vector alone resulted in the accumulation of GFP in the nucleus and cytoplasm (Figure 6A). In contrast, the full-length GbX-GFP construct are localized in an intracellular domain (probably the golgi apparatus) and in the cell membrane. The same localization was found for fusion-constructs lacking the last 16 amino acids of the C-terminus of GbX (GbXΔC) (Figure 6B, C). Immunostaining experiments using the GbX antibody showed overlapping signals for GFP and GbX in both cellular compartments (Figure S4).

In further transfection experiments, three mutant constructs were examined along with the GbXΔC construct. The G2A mutant- GFP-fusion protein, which is not myristoylated but palmitoylated, showed strong staining in the nucleus, but a weak membrane association was still detectable (Figure 6D). The palmitoylation-deficient C3S mutant was localized exclusively in the nucleus (Figure 6E). No membrane association was detected for this construct. The nonacylatable AS mutant, which neither carries the myristoylation nor the palmitoylation sites, was largely found in the nucleus and diffusively in the cytoplasm, thereby being indistinguishable from cells expressing GFP only (Figure 6F).

## Discussion

### Heme reactivity in GbX

Absorbance spectra of GbX were very similar to those reported for Ngb and Cygb [12], [23], indicating that this globin is hexacoordinate when in the absence of external ligands and that it forms a stable ferrous oxy complex in air, similar to Cygb [23]. O_2_ equilibria indicate Hill coefficients above unity and the existence of cooperativity, suggesting that – at least *in vitro* – GbX likely forms a dimer or tetramer, as also found for Cygb. A dimeric GbX is corroborated by a band at ∼36 kDa in SDS-PAGE (Figure S1, S2). O_2_ binding experiments reveal that the affinity for O_2_ decreases when thiols are in the reduced state (i.e. with DTT added), which suggests that heme reactivity is regulated by thiol oxidation state. As samples in the absence of DTT did not exclusively contain protein having the internal disulfide bridge (Figure S2), the increase in P_50_ of GbX attributable to the formation of the S-S bond may actually be higher than 4.0-fold. *D. rerio* GbX contains three Cys residues. Because Cys3 is palmitoylated and involved in membrane-association, disulfide bond formation most likely occurs between CysC4 and CysG14. These residues are conserved among fish GbX [17], suggesting that disulfide bond formation may to be critical for correct folding of GbX into a functional conformation.

### GbX is a neuronal protein in zebrafish

In previous studies using goldfish, a broad expression pattern of GbX in various non-neuronal tissues was observed [17]. By contrast, GbX was preferentially expressed in the brain and eye of the clawed frog *X. laevis* [16]. We identified high amounts of GbX mRNA in eyes and brain, which is consistent with the detection of GbX protein in selected brain and eye areas of *D. rerio*. We therefore conclude that the localization of GbX is likely conserved in fishes and amphibians. Whether the divergent pattern in goldfish is a unique feature of that species (possibly associated with its hypoxia tolerance) remains to be demonstrated.

In contrast to Ngb, which is apparently present in all nerve cells of the central and peripheral nervous systems [3], [28], GbX is confined to specific regions of the brain and retina. Interestingly, these parts of the central nervous system are associated with the visual or olfactory systems, respectively. In the zebrafish brain, strong GbX-staining was found in the oculomotor nucleus (NIII) and in nerves with axons innervating muscles that control the movements of the eye. The *n. oculomotorius* exits the brain ventrally and passes the hypothalamus, which may explain the immunostaining in this region (Figure 4A, C). In sagittal sections the hypothalamic *corpus mamillare* and *fasciculus retroflexus* exhibit prominent staining as well. The mamillary body (*c. mamillare*) is a pair of nuclei that receives and relays olfactory impulses. The *f. retroflexus* is a fiber tract that connects the habenula with the midbrain and hindbrain (Figure 4C) [29].

The vertebrate retina is composed of three layers of nerve cell bodies (outer and inner nuclear layer, ganglion cell layer) and two layers of synapses (inner and outer plexiform layer) [30]. GbX is localized in the ganglion cell layer (GCL) of the retina, which contains the nuclei of the ganglion cells and some displaced amacrine cells. The ganglion cells project visual signals from the photoreceptors to the *tectum opticum*, which represents the major visual center in teleosts. In summary, GbX appears to be associated mainly with neurons of the sensory system.

### GbX is likely attached to the membrane via S-palmitoylation and N-myristoylation

N-terminal lipid attachment results in association of the acylated protein with the cytoplasmic side of the membrane [31]. Our results suggest that GbX is indeed myristoylated at Gly2 and palmitoylated at Cys3. Myristoylation is a covalent and irreversible attachment of the fatty acid myristate (C14∶0), which is cotranslationally catalyzed by N-myristoyltransferase. This enzyme recognizes an N-terminal Gly, which is exposed by removal of the initiator Met (Figure 5) [32]. Unlike myristoylation, palmitoylation is reversible and therefore plays a role in regulatory functions, subcellular trafficking and localization [31], [33]. Palmitate (C16∶0) is posttranslationally attached to the protein by multiple enzymes. Myristoylation of GbX is essential for membrane localization, whereas palmitoylation is required for full association. A small proportion of GbX protein appears to be palmitoylated and localized at the membrane even in the absence of a prior myristoylation (Figure 6D). The complete lack of acylation, as in the GbX-mutant constructs, resulted in an accumulation of the GFP-tagged protein in the nucleus. Our results thus indicate that both lipid modifications are necessary for correct subcellular localisation of GbX.

Hb, Mb, and Ngb of vertebrates are proteins located in the cytoplasm. Cygb is a cytoplasmic protein in fibroblasts and related cells, but partly resides in the nucleus of some neurons [11]. Membrane-bound globins had been unknown in vertebrates, but have previously been reported in bacteria [19], [34], [35] and such a protein has also been identified in the gills of the green shore crab, *Carcinus maenas* [20]. Although the crab globin harbors an N-terminal N-myristoylation site (but no palmitoylation site), it is evolutionary not related to GbX, suggesting convergent evolution of membrane attachment in eukaryotes. To the best of our knowledge, GbX is therefore the first example in vertebrates where a globin is attached to the membrane. Nevertheless, membrane association of globins may be more widespread in animals than currently acknowledged and hint to a common but still poorly defined function of globins.

### Functional implications

Hb and Mb are respiratory proteins involved in transport or storage of O_2_. Although the O_2_-affinity values of recombinant GbX are within the range observed in typical tissue globins, the membrane-association of GbX renders a true respiratory function highly unlikely. Bacterial membrane-hemoglobins are either associated with the cell membrane or are located in the periplasmic space. It has been suggested that these globins may enhance the flux of O_2_ to a terminal oxidase of the respiratory chain, especially under hypoxic conditions, or that they may protect the terminal oxidase from reactive oxygen or nitrogen species (ROS/RNS) [19], [34], [35]. Because there is no respiratory chain in the cell membrane of eukaryotes, any such role of GbX can be excluded. In addition, it has been proposed that some bacterial membrane-globins may preserve the integrity of membrane lipids by reducing peroxides that had been formed in response to ROS stress [34]. Such a function is in fact conceivable for GbX. This may further explain the association of GbX with the sensory nerve system, which is known to have high metabolic rates and thus high ROS production. As Cys residues are a target for *in vivo* H_2_O_2_, this feature may link Cys redox state of GbX with yet unidentified heme reactivity *in vivo*.

Alternatively, GbX may be involved in some type of signal transduction process, either directly as an O_2_ sensor or as a binding partner in signal cascades. This hypothesis is in line with the acylation and membrane-association of GbX. Several proteins involved in signal transduction are dually acylated [36], such as non-receptor tyrosine kinases [37], the Gαi family [38] and Ca^2+^-dependent protein kinases [39]. GbX may act as O_2_-sensing protein, provided that a reducing system exists to maintain the protein in the ferrous form. Although several heme-containing proteins either of mitochondrial (e.g. cytochrome oxidase) or non-mitochondrial origin have been described as putative O_2_ sensors (e.g. nitric oxide synthase, NADPH oxidase, oxygen sensitive K^+^ channels), in most cases the signal transduction mechanism is unknown [40], [41]. In some prokaryotes, O_2_ is detected by globin-coupled sensors which consist of a regulatory globin-like heme-binding domain and a linked transducer domain [42], [43]. Recently, is has been proposed that vertebrate Cygb oxidizes lipids, thereby generating signaling lipids under oxidative conditions [44]. In analogy, a signaling function is also conceivable for GbX.

Although additional studies clearly are required to elucidate the true physiological role of GbX, the identification of this acylated, membrane-bound globin adds a new and unexpected complexity to the family of vertebrate globins. The fact that GbX has been lost in “higher vertebrates” (*i.e.* Amniota) must be taken into account when explaining its function.

## Supporting Information

Figure S1 **Purification of recombinant GbXΔNC.** GbXΔNC was expressed in *E. coli* BL21(DE3)pLysS host cells and purified using a three-step protocol consisting of ammonium sulfate precipitation, ion-exchange and size exclusion chromatography. M: Molecular mass marker; lane 1: *E. coli* proteins before induction with IPTG; lane 2: Proteins after induction with IPTG; lane 3: ammonium sulfate precipitation (60 to 80%); lane 4: pooled peak fractions of ion-exchange chromatography; lane 5: pooled peak fractions of size exclusion chromatography. The arrow indicates the position of GbX at the expected mass of ∼18 kDa.
(TIF)

Figure S2 **PAGE-analysis of recombinant GbXΔNC.** SDS-PAGE (10–15%) in the absence (−) and presence (+) of DTT (A) and isoelectrofocusing (IEF) of *D. rerio* GbX on ultrathin polyacrylamide gel (pH 3–9) (B) Lane 1 in (A) refers to GbX, lanes 2, 3 and M refer to, respectively, carbonic anhydrase (30 kDa), myoglobin (17.5 kDa) and standard proteins (low-range molecular weight markers with indicated molecular masses). Lanes M, 1 and 2 in (B) refer to, respectively, broad-range pI markers standard, and of GbX after and before prolonged exposure to air at room temperature. The isoelectric points of protein markers (left) and of GbX (right) are indicated.
(TIF)

Figure S3 **Western Blot analysis of GbX-GFP fusion constructs.** 3T3 cells transiently transfected with wild type GFP, full length (GbX) and C-terminally truncated (GbXΔC) GbX. Fusion constructs were detected by a specific anti-GFP antibody (Abcam). GFP: wild type GFP without GbX, GbX: full length GbX, GbXΔC: C-terminally truncated GbX, G2A: nonmyristoylatable mutant, C3S: nonpalmitoylatable mutant, AS: nonacylatable mutant. Protein marker (Fermentas) is indicated (M), expected masses of fusion constructs are 50 (GbX) and 47 kDa (GbXΔC, G2A, C3S, AS), respectively. Expression of all constructs is detectable (arrow).
(TIF)

Figure S4 **Co-staining of GbX and GFP in 3T3 cells.** Immunofluorescence studies of 3T3 cells transfected with GbXΔC-GFP and stained with anti-GbX antibody. GbX is clearly localized at the cellular membrane (A & B) and in intracellular membranes (D & E). Merged figures demonstrate co-localization of GbX and GFP (C & F).
(TIF)
