# Supplementary figures and images for: A Membrane-Bound Vertebrate Globin
Source: PLoS One. 2011 Sep 20;6(9):e25292. doi: 10.1371/journal.pone.0025292 (PMC3176823; doi:10.1371/journal.pone.0025292)

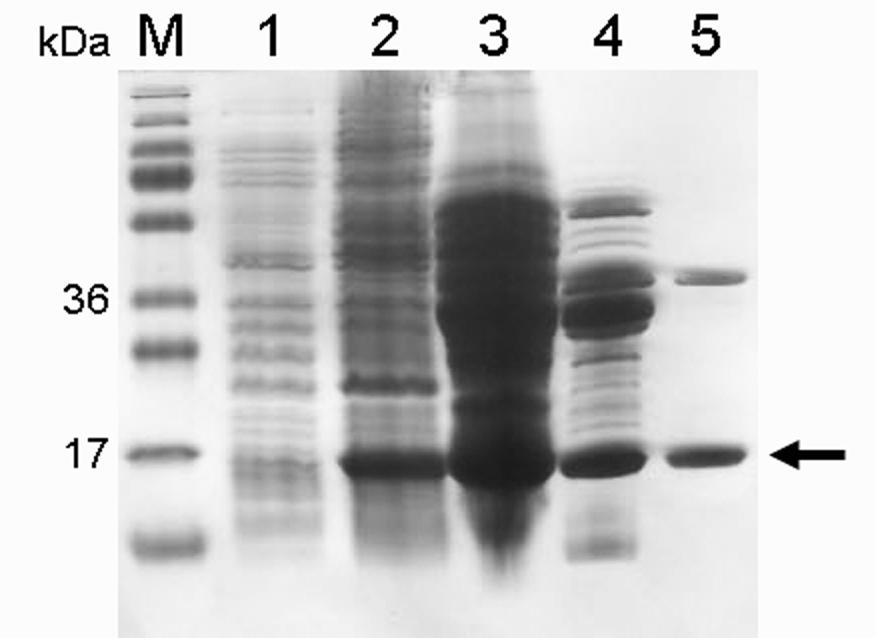

Supplement: Figure S1 — Purification of recombinant GbXΔNC. GbXΔNC was expressed in E. coli BL21(DE3)pLysS host cells and purified using a three-step protocol consisting of ammonium sulfate precipitation, ion-exchange and size exclusion chromatography. M: Molecular mass marker; lane 1: E. coli proteins before induction with IPTG; lane 2: Proteins after induction with IPTG; lane 3: ammonium sulfate precipitation (60 to 80%); lane 4: pooled peak fractions of ion-exchange chromatography; lane 5: pooled peak fractions of size exclusion chromatography. The arrow indicates the position of GbX at the expected mass of ∼18 kDa. (TIF) [file pone.0025292.s001.tif]

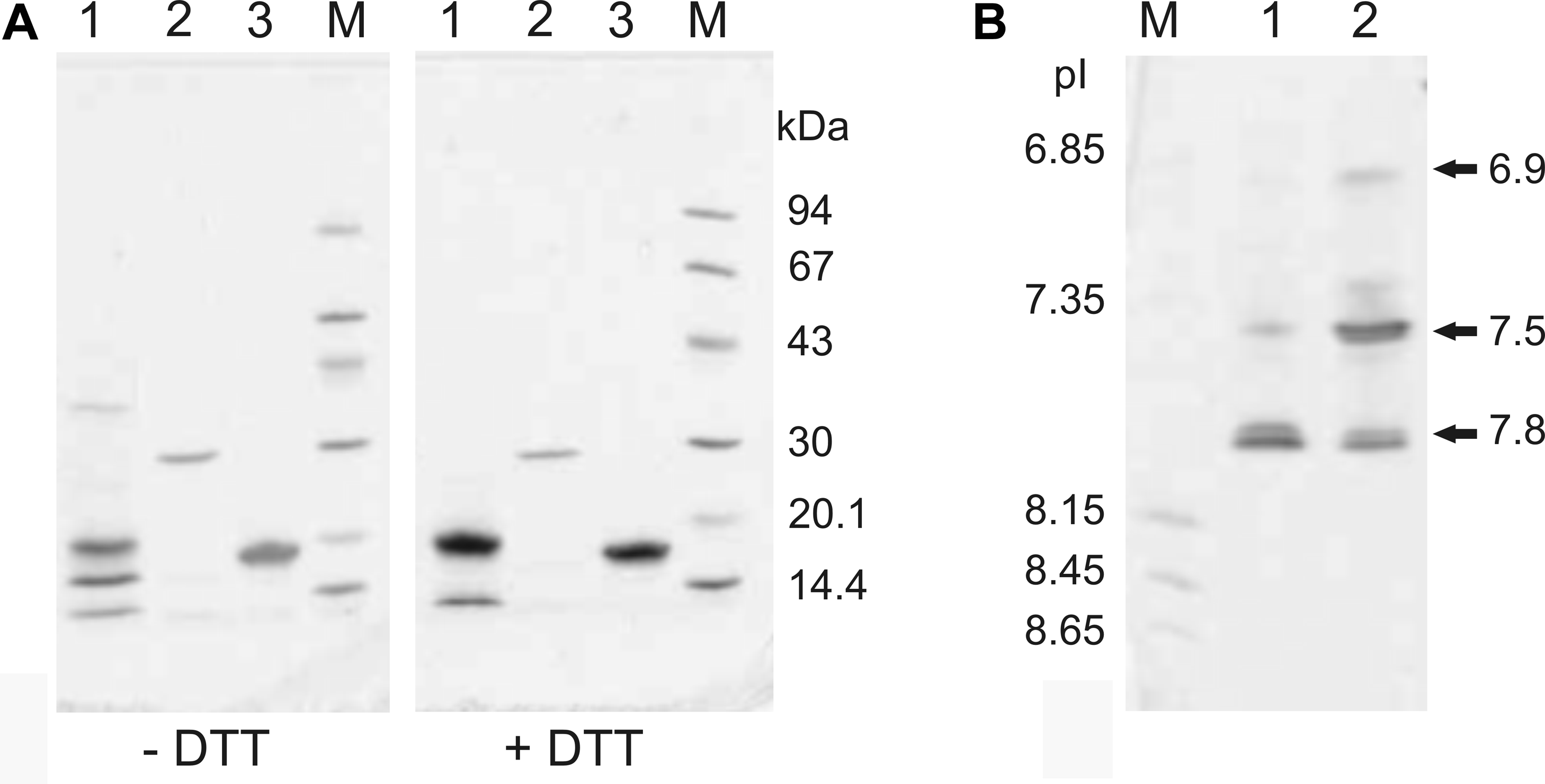

Supplement: Figure S2 — PAGE-analysis of recombinant GbXΔNC. SDS-PAGE (10–15%) in the absence (−) and presence (+) of DTT (A) and isoelectrofocusing (IEF) of D. rerio GbX on ultrathin polyacrylamide gel (pH 3–9) (B) Lane 1 in (A) refers to GbX, lanes 2, 3 and M refer to, respectively, carbonic anhydrase (30 kDa), myoglobin (17.5 kDa) and standard proteins (low-range molecular weight markers with indicated molecular masses). Lanes M, 1 and 2 in (B) refer to, respectively, broad-range pI markers standard, and of GbX after and before prolonged exposure to air at room temperature. The isoelectric points of protein markers (left) and of GbX (right) are indicated. (TIF) [file pone.0025292.s002.tif]

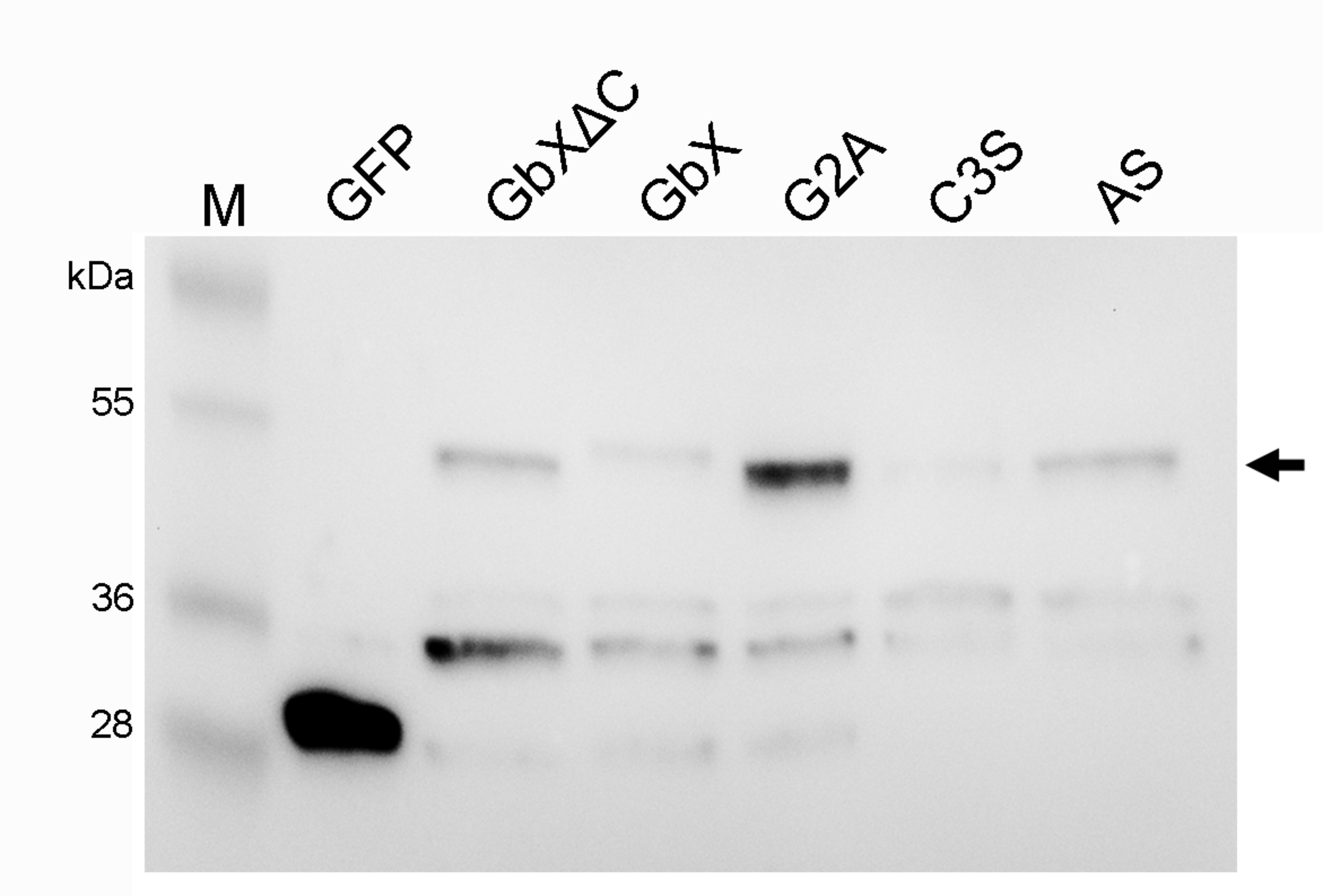

Supplement: Figure S3 — Western Blot analysis of GbX-GFP fusion constructs. 3T3 cells transiently transfected with wild type GFP, full length (GbX) and C-terminally truncated (GbXΔC) GbX. Fusion constructs were detected by a specific anti-GFP antibody (Abcam). GFP: wild type GFP without GbX, GbX: full length GbX, GbXΔC: C-terminally truncated GbX, G2A: nonmyristoylatable mutant, C3S: nonpalmitoylatable mutant, AS: nonacylatable mutant. Protein marker (Fermentas) is indicated (M), expected masses of fusion constructs are 50 (GbX) and 47 kDa (GbXΔC, G2A, C3S, AS), respectively. Expression of all constructs is detectable (arrow). (TIF) [file pone.0025292.s003.tif]

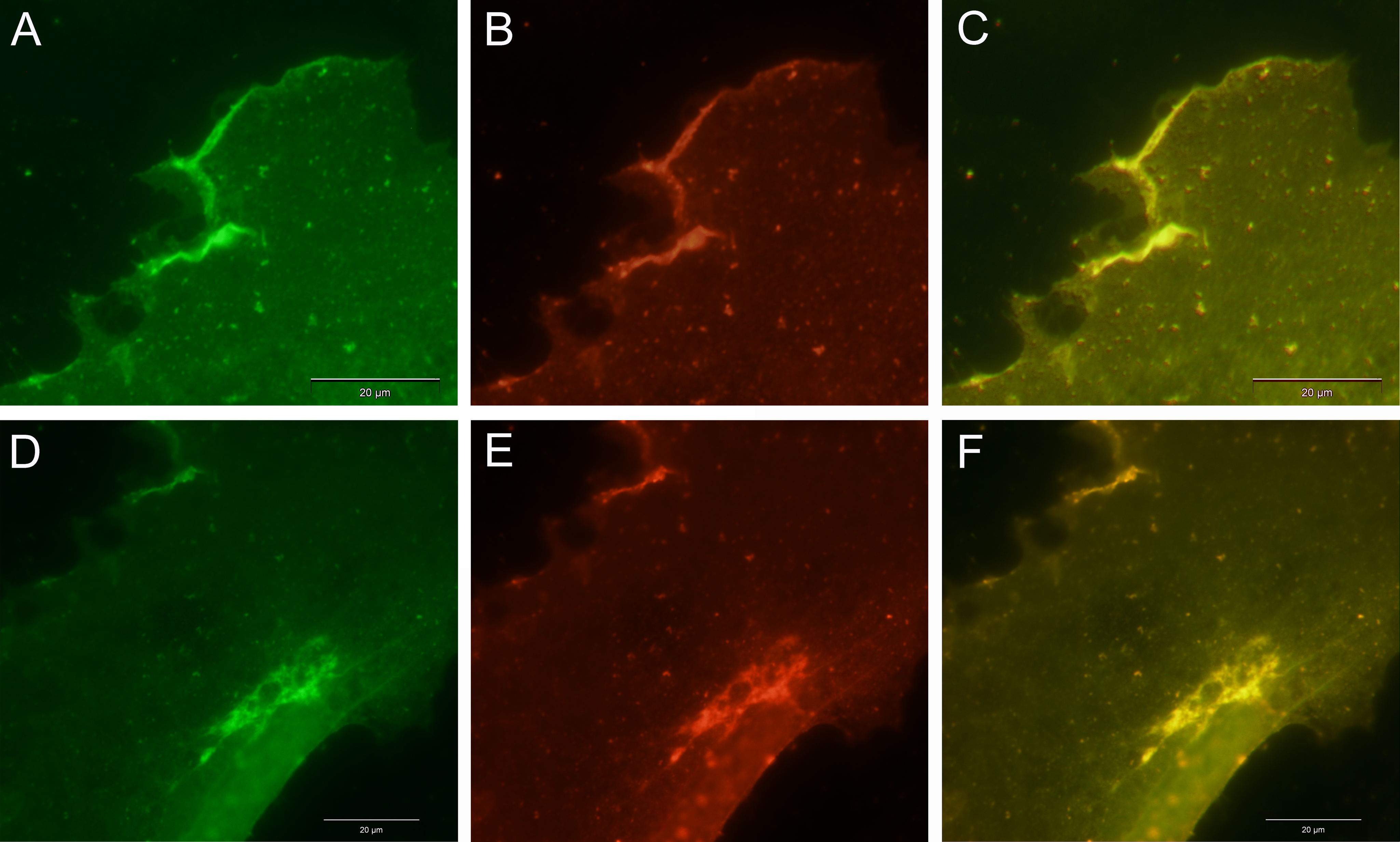

Supplement: Figure S4 — Co-staining of GbX and GFP in 3T3 cells. Immunofluorescence studies of 3T3 cells transfected with GbXΔC-GFP and stained with anti-GbX antibody. GbX is clearly localized at the cellular membrane (A & B) and in intracellular membranes (D & E). Merged figures demonstrate co-localization of GbX and GFP (C & F). (TIF) [file pone.0025292.s004.tif]
